# Challenges in prompt identification and surgical correction of Marfan Syndrome aortic disease in a middle-income country: a case series study

**DOI:** 10.1186/s13019-024-02793-w

**Published:** 2024-06-07

**Authors:** Alejandro Velandia-Sánchez, Camilo A. Polanía-Sandoval, Julián Senosiain-González, José V. Álvarez-Martínez, Sebastian Gallo-Bernal, Juan G. Barrera-Carvajal, Juan P. Umana, Jaime Camacho-Mackenzie

**Affiliations:** 1 Department of Cardiovascular Surgery, Fundación Cardioinfantil-Instituto de Cardiología, Bogotá, Colombia; 2 Vascular and Endovascular Surgery Research Group, Fundación Cardioinfantil-Instituto de Cardiología, Cra 13B No. 161-85 Torre I Piso 8, Bogotá, 110131 Colombia; 3 School of Medicine and Health Sciences, Universidad del Rosario, Bogotá, Colombia; 4 Division of Radiology, Massachusetts General Hospital, Boston, MA USA

**Keywords:** Marfan Syndrome, Connective tissue disorder, Aortic disease, Acute aortic syndromes, Aortic aneurysm, Aortic dissection, Emergent approaches

## Abstract

**Background:**

Marfan Syndrome is an autosomal dominant disease caused by pathogenetic variants in the FBN1 gene. The progressive dilatation of the aorta and the potential risk of acute aortic syndromes influence the prognosis of these patients. We aim to describe population characteristics, long-term survival, and re-intervention patterns in patients who underwent aortic surgery with a previously confirmed clinical diagnosis of Marfan Syndrome in a middle-income country.

**Methods:**

A retrospective single-center case series study was conducted. All Marfan Syndrome patients who underwent aortic procedures from 2004 until 2021 were included. Qualitative variables were frequency-presented, while quantitative ones adopted mean ± standard deviation. A subgroup analysis between elective and emergent procedures was conducted. Kaplan-Meier plots depicted cumulative survival and re-intervention-free. Control appointments and government data tracked out-of-hospital mortality.

**Results:**

Fifty patients were identified. The mean age was 38.79 ± 14.41 years, with a male-to-female ratio of 2:1. Common comorbidities included aortic valve regurgitation (66%) and hypertension (50%). Aortic aneurysms were observed in 64% without dissection and 36% with dissection. Surgical procedures comprised elective (52%) and emergent cases (48%). The most common surgery performed was the David procedure (64%), and the Bentall procedure (14%). The in-hospital mortality rate was 4%. Complications included stroke (10%), and acute kidney injury (6%). The average follow-up was 8.88 ± 5.78 years. Survival rates at 5, 10, and 15 years were 89%, 73%, and 68%, respectively. Reintervention rates at 1, 2.5, and 5 years were 10%, 14%, and 17%, respectively. The emergent subgroup was younger (37.58 ± 14.49 years), had the largest number of Stanford A aortic dissections, presented hemodynamic instability (41.67%), and had a higher requirement of reinterventions in the first 5 years of follow-up (*p* = 0.030).

**Conclusion:**

In our study, surveillance programs played a pivotal role in sustaining high survival rates and identifying re-intervention requirements. However, challenges persist, as 48% of the patients required emergent surgery. Despite not affecting survival rates, a greater requirement for reinterventions was observed, emphasizing the necessity of timely diagnosis. Enhanced educational initiatives for healthcare providers and increased patient involvement in follow-up programs are imperative to address these concerns.

## Background

Marfan syndrome (MFS) is a multisystem autosomal dominant connective tissue disorder associated with mutations in the gene for fibrillin-1 (FBN1) [1, 2]. MFS is the most common inherited connective tissue disorder, with an estimated birth incidence of 1 per 14.217 individuals [3–5].

Amongst the various phenotypic features of MFS, aortic disease represents the most concerning manifestation, due to the high risk of developing life-threatening events, such as acute aortic syndromes, that account for the decreased life expectancy observed in these patients [6–8]. An ascending aortic aneurism involving the aortic root is the hallmark Aortic Disease in MFS; this progressive dilation of the aorta can precipitate an aortic rupture, acute type A aortic dissection, aortic regurgitation, or in some cases these complications may coexist [6, 9, 10]. Nevertheless, dilation or dissection of the aortic arch, and descending thoracic or abdominal aorta can also occur [11, 12].

Prompt identification of MFS can significantly impact the life expectancy of these patients [2]. It has been demonstrated that a strict follow-up subsequent to the MFS diagnosis is essential to determine the requirement for prophylactic aortic root interventions, directed to avoid complications such as Acute Aortic Dissection (ADD), as it is related to lower long-term survival and a higher rate of re-interventions [2]. Likewise, accurate interventions such as lifestyle adjustments and pharmacological therapy with antihypertensive agents (beta-blockers and/or angiotensin receptor blockers) can reduce hemodynamic stress on the aortic wall, and therefore a reduction in the aortic root growth rates [2].

Current interventional treatment options include different techniques, including composite valve graft replacement of the aortic valve and ascending aorta (Bentall procedure), VSRR with reimplantation of the coronary arteries (David procedure), and less often, endovascular or hybrid interventions [9, 13, 14].

The current guidelines emphasize monitoring the aortic root and ascending aorta in patients with MFS. According to the American Heart Association (AHA) recommendations, elective repair is advised when the diameter reaches 5.0 cm (Class 1, Level C-LD). Nevertheless, key risk factors for aortic complications have been identified. These factors include a family history of aortic dissection, rapid aortic growth (≥ 0.3 cm/year), diffuse dilation of the aortic root and ascending aorta, and marked vertebral arterial tortuosity. The guidelines suggest elective aortic root surgery after reaching 4.5 cm when these risk factors are present (Class 2, Level B-NR) [15].

Additionally, dilation of other anatomical locations of the aorta is more likely to occur after aortic root replacement or previous dissection. For aneurysms in other aortic segments (arch, descending, or abdominal), operative intervention is considered reasonable when the aortic diameter reaches ≥ 5.0 cm for patients at acceptable operative risk or with a long life expectancy (Level 2a, Class C-EO) [15].

However, optimal surgical timing and surgical outcomes are a current concern. These guideline recommendations are based on observational studies and experts’ opinions with mainly short-term and mid-term results [2]. Moreover, the literature on the adequate treatment of MFS aortic disease in Latin America is even scarcer and limited [16, 17].

Therefore, we aim to describe population characteristics, long-term survival, and re-intervention patterns in patients who underwent aortic surgery with a previously confirmed clinical diagnosis of MFS within a high-level experienced cardiovascular institution in a middle-income country between 2004 and 2021.

## Methods

This is an observational, retrospective case series study, from January 2004 to December 2021 3534 procedures involving the aortic valve and/or the aorta were performed at a fourth-level institution in Colombia. All patients who underwent aortic surgery at any anatomic location and a clinical diagnosis of MFS based on the Ghent II criteria were included. Exclusion criteria were patients under 18 years of age, incomplete data, and patients in whom intervention was performed outside our institution. The main outcomes of the study were to describe population characteristics, long-term survival, and re-intervention patterns.

MFS diagnosis was made based on Ghent II criteria without a genetic identification of the FBN1 mutation. Nevertheless, while not all patients presented with every individual feature, the combination of specific manifestations, including aortic root dilatation, ectopia lentis, a systemic score of ≥ 7, and a family history of MFS, fulfilled the criteria for the diagnosis of MFS for each patient according to the Ghent II criteria [18].

Sociodemographic, preoperative, intraoperative, postoperative, and follow-up variables were obtained by electronic and physical chart review. The study specifically targeted those patients who developed aortopathies, comprising aortic aneurysm, aortic dissection, or both at any anatomic location. Throughout the 17-year retrospective study, elective and emergent procedures were considered, and the distinction between these interventions was maintained. Emergent procedures were defined as surgical interventions needed 24 h from hospital admission.

An overall descriptive analysis was conducted. For quantitative variables, the Shapiro-Wilk test was used to assess their distribution. Variables with a normal distribution were analyzed using mean and standard deviation, while those with a non-parametric distribution were analyzed using the median and interquartile range (IQR) (25th-75th percentile). Categorical variables were analyzed using relative and absolute frequencies.

For the follow-up process, the combination of clinical assessments with government data to track out-of-hospital mortality was carried out. 5 patients were lost in follow-up, 2 due to in-hospital mortality, and 3 due to barriers in the follow-up appointment, as it can be assigned to another institution due to our country’s healthcare system or because the patient did not attend. We incorporated Kaplan-Meier plots to visually illustrate survival and freedom from the first reintervention. This analytical approach allowed for a dynamic representation of these interventions over the 15-year follow-up period.

Additionally, we conducted a subgroup analysis between elective and emergent procedures. A descriptive analysis was made to characterize the distinctive features of each subgroup, using the same parameters described in the overall analysis. Kaplan-Meier plots were also used. Furthermore, a Log-rank (Mantel-Cox) test was applied.

Data analysis was conducted using Jamovi (Version 2.3) [computer software]. Figures were created with BioRender.com and edited with Adobe Photoshop (Version 5.3 for iPad).

## Results

Fifty patients met the inclusion criteria. The mean age was 38.79 ± 14.41 years, ranging from 18 to 70 years. Sixteen patients were female (32%) and thirty-four were male (68%), resulting in a sex ratio of 2:1 (Male/Female). Body mass index (BMI) showed overweight (22%) and obesity (4%). The most frequent comorbidities were aortic valve regurgitation (66%), hypertension (50%), mitral valve regurgitation (30%), and chronic heart failure (24%) (Table 1).

**Table 1:** Clinical and demographic characteristics of patients with MFS who underwent aortic procedures (*n*=50)

Characteristics of the population				Overall (*n*=50)		Elective (*n*=26)	Emergent (*n*=24)
Mean Age (years)				38.79 ± 14.41		43.65 ± 15.87	37.58 ± 14.49
Male				34 (68%)		16 (61.54%)	18 (75%)
Female				16 (32%)		10 (38.46%)	6 (25%)
Comorbidities							
Tobacco consumption				5 (10%)		0 (0%)	5 (20.83%)
Weight (BMI- kg/m2)		Low weight		4 (8%)		2 (7.69%)	2 (8.33%)
		Normal weight		33 (66%)		17 (65.38%)	16 (66.67%)
		Overweight		11 (22%)		5 (19.23%)	6 (25%)
		Obesity		2 (4%)		2 (7,69%)	0 (0)%
Dyslipidemia				5 (10%)		2 (7,69%)	3 (12.5%)
Diabetes mellitus				3 (6%)		2 (7,69%)	1 (4.17%)
Hypertension				25 (50%)		13 (50%)	12 (50%)
Cardiac arrhythmias				1 (2%)		1 (3.85%)	0 (0%)
Cronic Heart Failure				12 (24%)		10 (38.46%)	2 (8.33%)
Aortic valve pathology		Aortic valve regurgitation		33 (66%)		18 (69.23%)	15 (62.5%)
		Mild regurgitation		1 (4.5%)		0 (0%)	1 (4.16%)
		Moderate regurgitation		4 (18.2%)		0 (0%)	4 (16.67%)
		Severe regurgitation		17 (77.3%)		11 (42.3%)	6 (25%)
		Aortic valve stenosis		1 (2%)		1 (3.85%)	0 (0%)
Mitral valve pathology		Mitral valve regurgitation		15 (30%)		11 (42.31%)	4 (16.67%)
		Mitral valve stenosis		0 (0%)		0 (0%)	0 (0%)
Coronary artery disease				9 (18)%		5 (19.23%)	4 (16.67%)
Previous cardiac surgery				7 (14%)		2 (7,69%)	5 (20.83%)
Peripheral artery disease				5 (10%)		2 (7,69%)	3 (12.5%)
Chronic obstructive pulmonary disease				3 (6%)		3 (11.54%)	0 (0%)
Chronic kidney disease				3 (6%)		3 (11.54%)	0 (0%)

Subgroup descriptive analysis between elective and emergent aortic procedures is also shown. Data is presented as mean (SD) or frequencies (percentage %)

*BMI* Body Mass Index

**Fig. 1:**
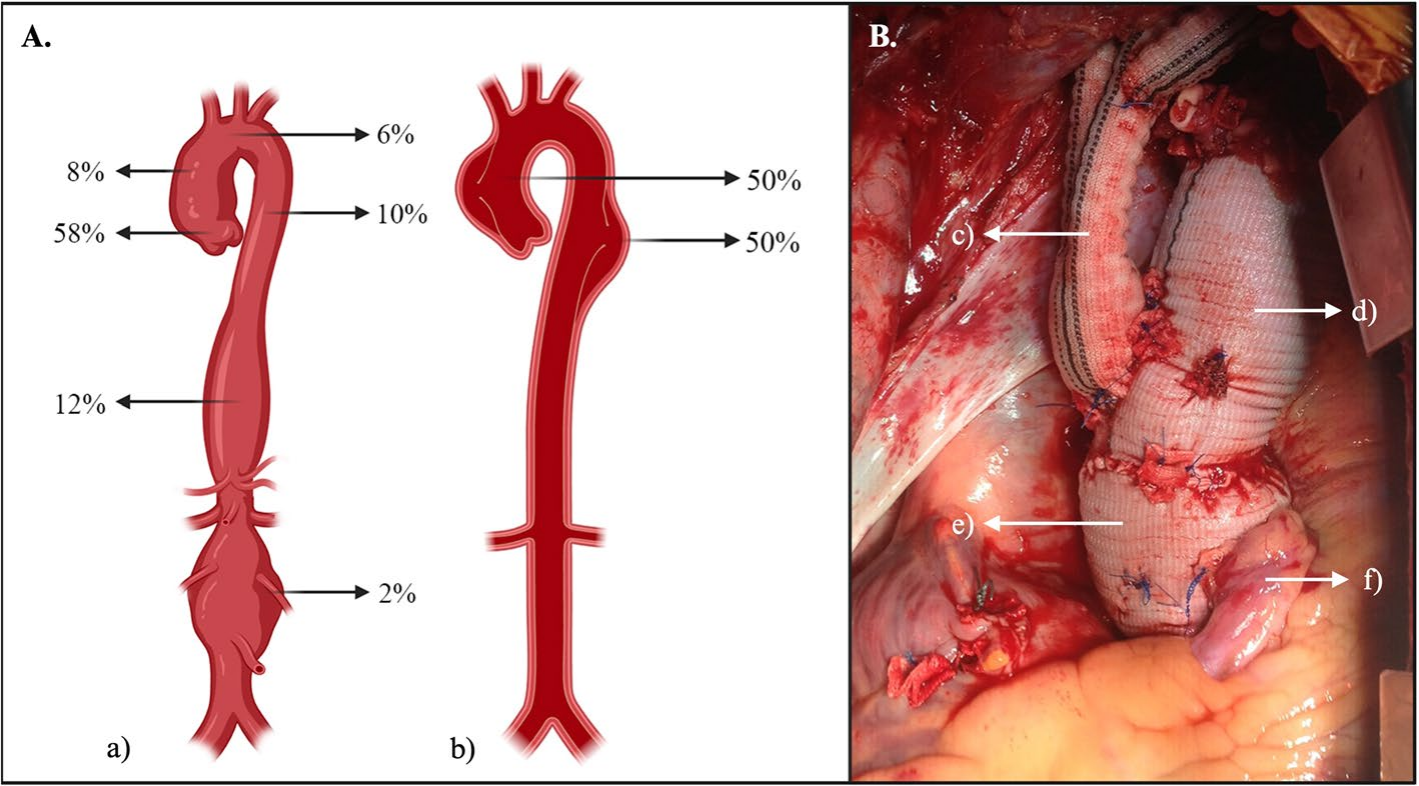
**A**. Anatomical distribution of aortopathies presented. a) Anatomical distribution and frequencies (percentage %) of aneurysm location. b) Anatomical distribution and frequencies (percentage %) of aortic dissection according to Stanford classification. **B**. Intraoperative image of a patient with MFS who underwent a one-stage David procedure and aortic hemiarch repair. c) Reimplantation of the supra-aortic vessels. d) Aortic hemiarch repair. e) David procedure. f) Coronary artery reimplantation

Aortic aneurysms without evidence of aortic dissection were found (64%), and aortic aneurysms with acute or chronic dissection were also present (36%), with an equal distribution between Stanford A and B classification (50%, respectively). The most common anatomical location was the aortic root (58%) with a mean size of 62.5 ± 19.6 mm, followed by the thoracoabdominal aorta (12%) with an average size of 66.4 ± 14.4 mm (Fig. 1 and Table 2).

**Table 2:** Aortic disease characteristics of patients with MFS that underwent aortic procedures (*n*=50)

Aortic disease characteristics			Overall (*n*=50)		Elective (*n*=26)	Emergent (*n*=24)
Aortic Aneurysm without dissection			32 (64%)		20 (76.92%)	12 (50%)
Aortic Aneurysm with dissection			18 (36%)		6 (23.08%)	12 (50%)
Stanford Classification		Stanford A	50%		1 (16.67%)	8 (66.67%)
		Stanford B	50%		5 (83.33%)	4 (33.33%)
Aneurysm location	Aortic root	Frequency	29 (58%)		10 (38.46%)	19 (79.17%)
		Mean Size (mm)	62.5 ± 19.6			
	Ascending aorta	Frequency	4 (8%)		4 (15.38%)	0 (0%)
		Mean Size (mm)	72.1 ± 14.5			
	Aortic arch	Frequency	3 (6%)		2 (7.69%)	1 (4.17%)
		Mean Size (mm)	68. 3 ± 12.7			
	Descending aorta	Frequency	5 (10%)		4 (15.38%)	1 (4.17%)
		Mean Size (mm)	56.0 ± 9.5			
	Thoracoabdominal aorta	Frequency	6 (12%)		4 (15.38%)	2 (8.33%)
		Mean Size (mm)	66.4 ± 14.4			
	Abdominal aorta	Frequency	1 (2%)		0 (0%)	1 (4.17%)
		Size (mm)	55.			
	Aortic root + thoracoabdominal	Frequency	1 (2%)		1 (3.85%)	0 (0%)
	Aortic root + abdominal	Frequency	1 (2%)		1 (3.85%)	0 (0%)

Subgroup descriptive analysis between elective and emergent aortic procedures is also shown. Data is presented as mean (SD) or frequencies (percentage %)

Surgery was elective in most cases (52%). Furthermore, emergent procedures represented a high proportion (48%). Among the patients undergoing surgery, the American Society of Anesthesiologists (ASA) score was predominantly class III (50%). The David procedure was the most common (64%) followed by the Bentall procedure (14%). 71.4% (5/7) of the Bentall procedure were performed with biological valves. The mean length of the intervention was 367.7 ± 96.2 min, blood transfusion was required for 40% of the patients, and a total of 74% of patients needed extracorporeal circulation (Table 3).

**Table 3:** Perioperative characterization of all initial surgical procedures, excluding reinterventions, in patients with MFS that underwent aortic procedures (*n*=50)

Surgical characteristics		Overall (*n*=50)	Elective (*n*=26)	Emergent (*n*=24)
Preoperative condition				
ASA Score	ASA I	0 (0%)	0 (0%)	0 (0%)
	ASA II	13 (26%)	12 (46.15%)	1 (4.17%)
	ASA III	25 (50%)	11 (42.31%)	14 (58.33%)
	ASA IV	12 (24%)	3 (11.54%)	9 (37.5%)
	ASA V	0 (0%)	0 (0%)	0 (0%)
Hemodynamic instability		10 (20%)	0 (0%)	10 (41.67%)
Hypovolemic shock		1 (2%)	0 (0%)	1 (4.17%)
Type of surgery performed				
Surgery performed	David	32 (64%)	17 (65.38%)	15 (52.5%)
	Bentall	7 (14%)	4 (15.38%)	3 (12.5%)
	Thoracoabdominal Aortic Aneurysm Repair	2 (4%)	0 (0%)	2 (8.33%)
	Abdominal Aortic Aneurysm Repair	1 (2%)	0 (0%)	1 (4.17%)
	Frozen Elephant Trunk	2 (4%)	1 (3.85%)	1 (4.17%)
	David + Aortic hemiarch repair	1 (2%)	1 (3.85%)	0 (0%)
	David + Full aortic arch repair	1 (2%)	1 (3.85%)	0 (0%)
	Bentall + Elephant trunk	2 (4%)	1 (3.85%)	1 (4.17%)
	David + Elephant trunk	1 (2%)	0 (0%)	1 (4.17%)
	David + Frozen elephant trunk	1 (2%)	1 (3.85%)	0 (0%)
Intraoperative characteritsics				
Mean length of intervention (min)		367.7 ± 96.2	298.5 ± 165.06	339.06 ± 143.66
Blood transfusion requirement		20 (40%)	6 (23.08%)	14 (58.33%)
Extracorporeal circulation		37 (74%)	14 (53.85%)	23 (95.83%)

Subgroup descriptive analysis between elective and emergent aortic procedures is also shown. Data is presented as mean (SD) or frequencies (percentage %)

*ASA* American Society of Anesthesiologists

The in-hospital mortality rate was 4%. The two patients had different approaches, one of them was an elective procedure and the other one was an emergent procedure. The elective procedure was a thoracoabdominal aortic open repair, who experienced postsurgical complications including coagulopathy, and multifactorial shock, leading to cardiac arrest. The second patient required an emergent procedure due to an ascending aortic rupture, a Tirone David procedure was performed. Unfortunately, despite efforts, this patient did not survive due to refractory cardiogenic shock after ECC retrieval.

The mean Intensive Care Unit (ICU) stay was 1.76 ± 1.61 days, and the average in-hospital stay was 11.14 ± 7.62 days. The most common complications were stroke (10%) followed by acute kidney injury (6%), and hypovolemic shock (4%) (Table 4).

**Table 4:** In-hospital postoperative characteristics of patients with MFS who underwent aortic procedures (*n*=50)

In-hospital postoperative characteristics		Overall (*n*=50)	Elective (*n*=26)	Emergent (*n*=24)
In-hospital mortality		2 (4%)	1 (3.85%)	1 (4.17%)
ICU stay		1.76 ± 1.61	1.58 ± 1.41	1.92 ± 1.78
In-hospital stay		11.14 ± 7.62	8.79 ± 7.10	13.30 ± 7.57
Stroke		5 (10%)	4 (15.38%)	1 (4.17%)
Cardiac arrest		1 (2%)	0 (0%)	1 (4.17%)
Hypovolemic shock		2 (4%)	1 (3.85%)	1 (4.17%)
Vocal cord paralysis		1 (2%)	1 (3.85%)	0 (0%)
Acute Kidney Injury		3 (6%)	1 (3.85%)	2 (8.33%)
Dialysis requirement		1 (2%)	0 (0%)	1 (4.17%)
Medular ischemia		0 (0%)	0 (0%)	0 (0%)
Surgcial site infection		0 (0%)	0 (0%)	0 (0%)
Sepsis		0 (0%)	0 (0%)	0 (0%)

Subgroup descriptive analysis between elective and emergent aortic procedures is also shown. Data is presented as mean (SD) or frequencies (percentage %)

*ICU* Intensive Care Unit

**Fig. 2:**
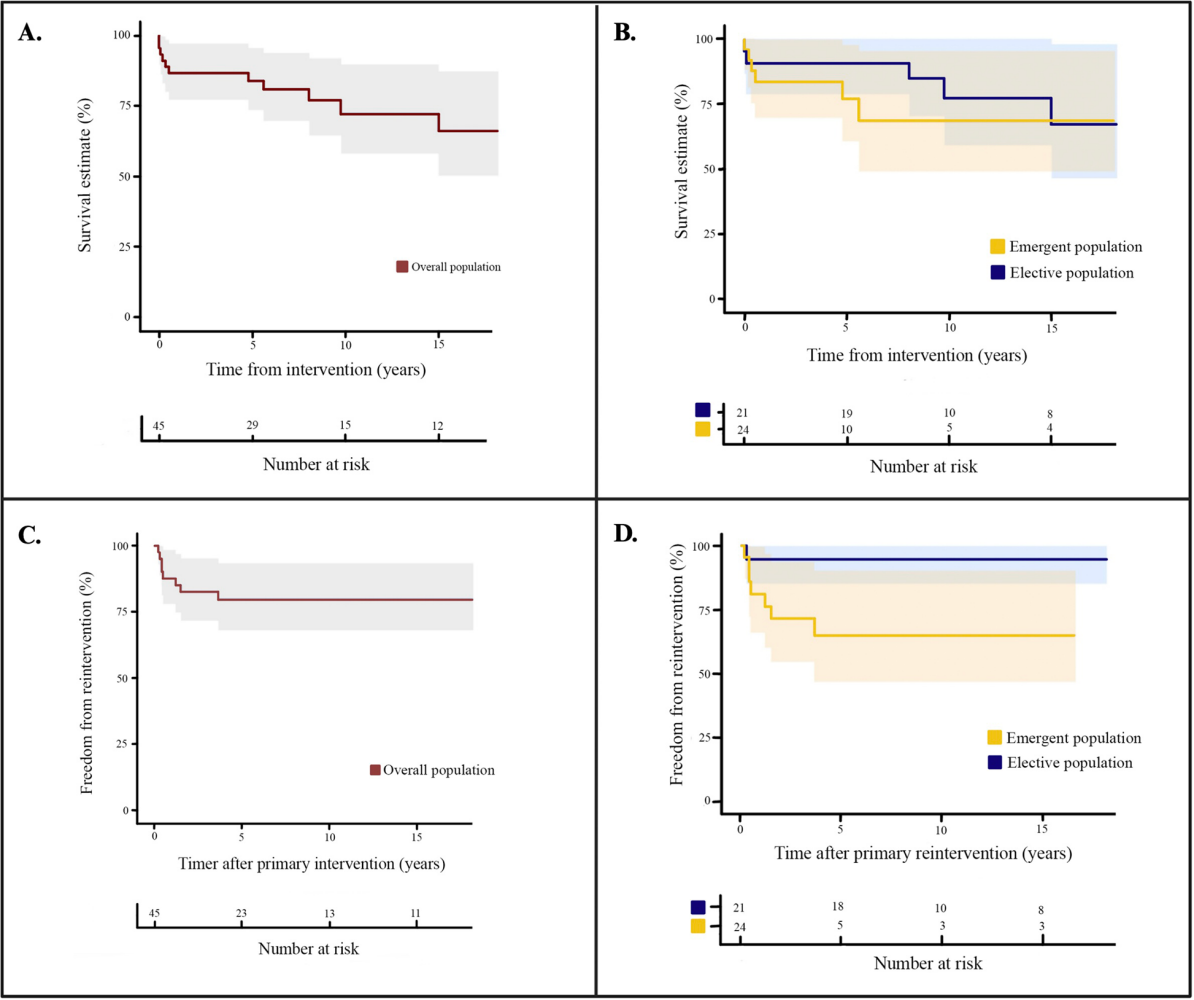
Survival analysis depicted through Kaplan-Meier plots spanning a 15-year follow-up. **A** Presents the overall survival of the 50 patients with MFS undergoing aortic surgery; (**B**) Conducts a subgroup survival analysis, distinguishing between elective and emergent aortic procedures; (**C**) Explores the overall freedom from reintervention for the included 50 patients; and (**D**) Provides a subgroup analysis, comparing the freedom from reintervention between elective and emergent aortic procedures

The average follow-up time was 8.88 ± 5.78 years. Survival rates at 5, 10, and 15 years were 89%, 73%, and 68%, respectively (Fig. 2-A). A total of 16% required at least one reintervention (*n* = 8), of which 3 patients required another reintervention and 2 patients required 2 more reinterventions (Table 5). The reintervention rates at 1, 2.5, and 5 years were 10%, 14%, and 17%, respectively (Fig. 2-C).

**Table 5:** Re-interventions involving aortic procedures in patients with MFS are outlined (*n*=8)

Patient number	First procedure performed	Reintervention #1		Reintervention #2		Reintervention #3	
		Cause	Procedure performed	Cause	Procedure performed	Cause	Procedure performed
1	David Procedure	Infrarenal Abdominal Aortic Aneurysm	Abdominal Aortic Aneursym Open Repair				
2	David + Aortic right hemiarch repair	Thoracoabdominal aortic aneurysm (T10 - Iliac Arteries)	Thoracoabdominal Aortic Aneurysm Open Repair	Aortic arch and Thoracic aneurysm	TEVAR + Supra-aortic debranching	Type B Aortic dissection	Aortic Left Hemiarch and Thoracic Open Repair
3	David procedure	Thoracic descending aortic aneurysm	TEVAR	Type IB endoleak	TEVAR	Type IA endoleak	Aortic left hemiarch repair
4	David + Aortic right hemiarch repair	Anastomotic Coronary Pseudoneurysm	Aortic Ascendent Open Repair	Type B Aortic Dissection and Thoracoabdominal aortic aneurysm	Thoracoabdominal Aortic Aneurysm Open Repair		
5	Thoracoabdominal Aortic Aneurysm Open Repair	Aortic root aneurysm and descending thoracic aneurysm	David + Frozen Elephant Trunk				
6	David + Frozen Elephant Trunk	Type IA endoleak	Full Aortic Arch Open Repair				
7	David Procedure	Type B Aortic Dissection and Thoracoabdominal aortic aneurysm	Thoracoabdominal Aortic Aneurysm Open Repair				
8	Bentall + Elephant trunk	Thoracoabdominal aortic aneurysm	Thoracoabdominal Aortic Aneurysm Open Repair				

The majority of these interventions are secondary to the natural progression of the disease, affecting other segments of the aorta, rather than as a direct result of complications secondary from the procedures. The reinterventions are not included in the Table 3

*TEVAR* Endovascular Repair or the Thoracic Aorta

### Subgroups analysis

We conducted a subgroup analysis based on the nature of the procedures performed, elective (*n* = 26) and emergent (*n* = 24). Variations between the patient ages were noted, as the patients in the emergent subgroup were younger (37.58 ± 14.49 years) and older in the elective subgroup (43.65 ± 15.87 years). Gender distribution revealed a higher proportion of males in both elective (61.54%) and emergent (75%) subgroups (Table 1).

The most common comorbidities in both subgroups were aortic valve regurgitation (69.23% and 62.5% for elective and emergent subgroups, respectively) and hypertension (50% for each subgroup). Notably, the elective subgroup exhibited a higher prevalence of chronic heart failure (38.46%), and mitral regurgitation (42.31%). Conversely, the emergent subgroup presented higher percentages of tobacco consumption (20.83%), with any of the patients presenting obesity, chronic obstructive pulmonary disease, or chronic kidney disease (Table 1).

The subgroup analysis showcased a predominant occurrence of aortic aneurysms without dissection in the emergent subgroup (50%), being Stanford A dissections the largest number of them (66.67%) unparalleled to the elective subgroup (16.67%). Despite the aortic root being the most common aneurysm anatomical location in both subgroups, the emergent subgroup had a greater proportion (79.17%) compared to the rest of the anatomical locations, while the elective subgroup also presented a great number of descending and thoracoabdominal aortopathies (15.38% in each anatomical location). The only patient who presented with an isolated abdominal aortic aneurysm was in the emergent subgroup (Table 2).

Surgical characteristics revealed diverse preoperative conditions. Patients in the elective subgroup were mostly classified as having an ASA score of II or III (46.15% and 42.31%, respectively). Nevertheless, the emergent subgroup had most of the patients classified with an ASA score of III (58.33%) and an important proportion with an ASA score of IV (37.5%). Hemodynamic instability and hypovolemic shock were only present in the emergent subgroup (41.67% and 4.17%, respectively). In terms of the type of surgery performed, the David procedure was the most common technique for both elective (65.38%) and emergent (52.5%) subgroups (Table 3).

Intraoperative characteristics highlighted variations in the mean length of intervention, and blood transfusion requirement with the emergent subgroup displaying a higher frequency in these parameters (339.06 ± 143.66 and 58.33% respectively) (Table 3).

In-hospital mortality was equally distributed in both subgroups with only 1 dead per subgroup. Mean ICU stay was similar in both subgroups (1.58 ± 1.41 and 1.92 ± 1.78 for the elective and emergent subgroups, respectively). However, the emergent subgroup presented a higher average in-hospital stay (13.30 ± 7.57). Most of the complications were similar between both subgroups, except for stroke which was predominant in the elective subgroup (15,38%) **(**Table 4). None of the subgroups presented spinal cord ischemia, surgical site infection, or sepsis.

No significant difference was established between the survival rates at any point between subgroups (*p* = 0.462) **(**Fig. 2-B**)**. However, the emergent subgroup had a significant difference in the requirement of reinterventions in the first 5 years of follow-up (*p* = 0.030). After the 5-year follow-up, a plateau was observed in both subgroups either for survival rates or freedom from reintervention (Fig. 2-D).

## Discussion

MFS is a connective tissue disorder characterized by the mutation of the FBN1 gene, which leads to impaired protein synthesis and incorporation of fibrillin-1 into the extracellular matrix, leading to all the clinical and pathological spectrum of MFS in the cardiovascular, ocular, and musculoskeletal tissue [19]. Individuals with MFS, along with those with other connective tissue disorders, often present with a higher susceptibility to aortic pathologies at a younger age [15]. In concordance, in our study, the mean age was 38.79 ± 14.41.

Aortic root dilation and type A aortic dissection are the main contributors to morbidity and mortality in MFS. Notably, patients with MFS are prone to developing aneurysms in the aortic root and ascending aorta early in life, experiencing a faster rate of aortic growth compared to those with sporadic aneurysms [15]. In our study, aortopathies could be identified in all anatomical locations of the aorta (Fig. 1). Consistent with the evidence, the majority were in the aortic root (58%).

**Fig. 3:**
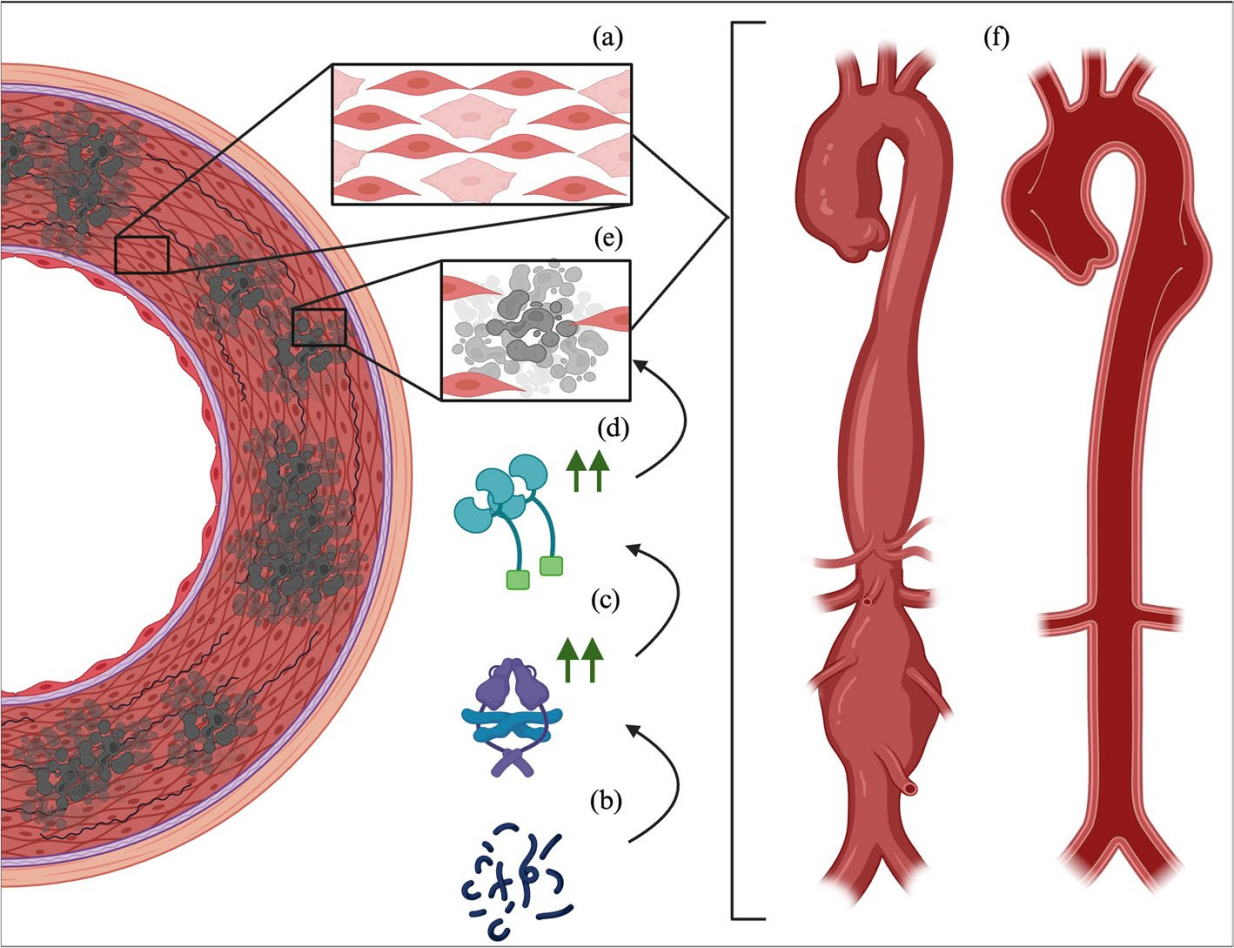
Illustration of the two-hit hypothesis in Marfan Syndrome-associated aortopathy: The initial hit (**a**) is characterized by the presence of immature smooth muscle cells in the aortic wall. Subsequently, the second hit unfolds through a sequential cascade: (**b**) involving aberrant fibrillin-1 and reduced normal fibrillin-1, (**c**) which triggers heightened intracellular production of TGF-B. This, in turn, leads to (**d**) increased proteinase activity causing extracellular matrix degradation, ultimately resulting in (**e**) smooth muscle cell apoptosis. Both pathways converge at (**f**) aneurysmal degeneration, heightening susceptibility to subintimal dissection, and acute aortic syndromes

Multiple theories regarding aortic arterial degeneration in MFS, have been proposed. The major role is guided toward smooth muscle cells (SMC) within the arterial wall. In the aortic root, the quantity of this type of cell is bigger due to the high pressures that it receives. It has been proposed that the inability of SMC to do the phenotypic switch between quiescent to proliferative state is related to the origin of multiple aortic pathologies. In MFS, histopathologic changes on the aortic root revealed cytolytic necrosis of the media, which can lead to the development of aneurysmal disease and/or aortic dissections. All this revealed a double-hit hypothesis for aortic disease and associated complications in MFS, in which the aortic root in these patients has abnormal immature SMC and low fibrillin-1 levels, leading to increased aortic vulnerability and subsequent aortopathies [19] (Fig. 3).

The surgical management of vascular manifestation associated with MFS is broad and depends on the type of aortopathy (aneurysmal degeneration, acute or chronic dissection and/or rupture), the anatomic location (most commonly the aortic root), and the patient’s clinical status.

Open surgical approaches might be preferred to patients with MFS mainly because of the complex and extensive involvement of the aorta (with the chance of also requiring the intervention of the aortic valve), the increased risk for endoleaks and endoprosthesis migration, and the young age of the patients that may face a lifetime risk of aortic complications [15]. However, endovascular and hybrid approaches have been reported in the literature [13, 14, 20–22]. In our case series, 88% of the procedures were performed with open approaches. The 12% corresponded to hybrid procedures, specifically the Frozen Elephant Trunk (FET) and its predecessor the Elephant Trunk (ET).

FET may be a great possibility for patients with MFS and involvement of the aortic arch and/or the descending aorta. It has the potential to address issues related to endoleaks and the migration of endoprostheses by securing the stent graft more proximally. This approach aims to reduce the occurrence of Type I and Type III endoleaks or the displacement of the stent-graft and provide an instrument for incoming surgeries if needed [20, 21]. The method involves accessing the aortic arch through a sternotomy and directly suturing the stent graft to facilitate a more secure fixation within the aortic arch repair [23]. The study published by Widenka et al. supported this argument, as 37 patients with MFS who underwent a FET were analyzed and at 5 years of follow-up, no aortic arch reinterventions were required [21]. Also, FET has shown that in patients with MFS and type A aortic dissection can effectively reduce the false lumen and stabilize the distal aorta [22].

In our study, the David procedure was the most frequent technique performed, accounting for 64% of all surgical procedures; followed by the Bentall procedure with 14%. A systematic review and meta-analysis by Burgstaller et al. revealed that patients with MFS undergoing a David procedure exhibited a favorable in-hospital mortality compared to those undergoing the Bentall procedure (OR 0.23; 95% CI 0.09–0.55, *P* = 0.001). Additionally, the David procedure demonstrated higher mid- and long-term survival rates (96.7% and 93.1%) compared to the Bentall procedure (86.4% and 82.6%) [24]. The only study we found in the Latin-American population, was published by Favoloro et al., who analyzed 54 patients who underwent surgical correction of the ascending aorta, being the Bentall procedure the most common (39 patients) and found a higher mortality rate for emergency surgery compared with elective surgery (*P* < 0.001) [17].

Despite this favorable evidence, the selection choice involves weighing various factors to individualize the approach to each patient’s condition and desires. Some of the advantages of the David procedure include the non-requirement of anticoagulation and the evidence shown previously, however, the patient should only have mild or no aortic regurgitation, an aortic root diameter less than 55 mm, and some evidence questions its durability, given the risk of developing or worsening aortic regurgitation at one year [25] On the other hand, the Bentall procedure offers an alternative since it replaces the aortic valve for life, in exchange for certain disadvantages such as permanent anticoagulation, increased risk of thromboembolic events, and/or endocarditis. Many times, the decision can also be made intraoperatively with a direct evaluation of aortic valvular fenestrations or calcifications [2]. Additionally, we identified that in our study chronic heart failure (CHF) rate is high among the cohort and this might be reflected in aortic valve damage and subsequent heart disease as 12 patients with CHF 10 had significant aortic valve regurgitation. This variable might be associated; however, more robust studies are needed to support this statement.

Regarding AAD, one of the biggest studies carried out by L. de Beaufort et al. reported important data on aortic dissection in patients with MFS compared with patients without this syndrome by using the International Registry on Acute Aortic Dissection. This study took data between January 1996 and May 2017, including almost 6,424 consecutive patients with AAD, from which 258 had MFS. They found that MFS-diagnosed patients tend to have an AAD at a lower age (38.2 ± 13.2 years) compared to adults without it (63.0 ± 14.0 years). In the same study, in both groups, type A Stanford dissection was more common than type B dissection (63.6% vs. 36.4%) [26]. Conversely, in our study, half of the patients presented with type A Stanford aortic dissection. This can be biased due to multiple reasons, including the limited sample size and the retrospective fashion of our study as discussed below. Nonetheless, the subgroup analysis showed that 90% of the type A Stanford aortic dissection were in the emergent subgroup, accounting for 66.67% of AAD of this subgroup aligning with the literature published.

Another important finding, that the study by L. de Beaufort et al. report is that patients with MFS who had a first surgery due to an AAD, more frequently required a reintervention compared to patients without MFS, reporting freedom from reintervention of 44.7% compared to 81.5% (*P* < 0.001) [26]. These results have been supported by another study, that also reports that patients treated for an initial AAD, are more likely to have any reintervention compared to patients who were intervened initially for an aneurysmal degeneration without AAD (*P* = 0.008) [27].

Accounting for all procedures, in our study, we found that 16% (*n* = 8) of patients required any type of reintervention, with reintervention rates at 1, 2.5, and 5 years of 10%, 14%, and 17%, respectively (Fig. 2-C). However, most of the interventions in our study are secondary to the natural progression of the disease, affecting other segments of the aorta, rather than as a direct result of complications secondary to the procedures. Only endoleaks and the anastomotic pseudoaneurysm can be derived from the procedures (Table 5). These results highlight the complexity and diversity of aortic pathology in MFS and show the need for individualized surgical approaches and proper follow-up programs.

Furthermore, the subgroup analysis showed that the emergent group had a higher requirement of reinterventions in the first 5 years of follow-up (*p* = 0.030) (Fig. 2-D). These results support the statement that MFS patients should be under rigorous surveillance programs as they are prone to needing any reintervention, at least for the first 5 years after the primary intervention, but this data must be confirmed to make clinical decisions.

The high frequency of procedures performed emergently (48%) and its relationship with a greater number of reinterventions are results that raise concern in our study. Although it has also been observed that patients with MFS who underwent first for emergent corrections first have in-hospital and higher long-term mortality [2, 17], this was not the case in our study, with survival rates at 5, 10, and 15 years were 89%, 73%, and 68%, respectively, and no significant difference between emergent and elective procedures (Fig. 2-B and D). Nevertheless, this implies the importance of prompt diagnosis and treatment of MFS.

A prompt diagnosis and adequate surveillance programs can impact the quality of life of patients with MFS. Some major reasons are the early detection and surgical treatment of aortopathies, which can impact on higher survival rates or freedom from reintervention, but also setting up proper medical therapy and educating on appropriate lifestyle modifications [2]. It is essential to implement improved educational programs for healthcare providers and encourage greater participation of patients in post-treatment programs to effectively tackle these issues in middle-income countries.

The present study can provide some insights into the population characteristics, long-term survival, and re-intervention patterns of surgical correction of MFS aortic disease, especially in Latin America where data is scarce. However, its retrospective and single-center design entails certain limitations that may impact the reliability and applicability of its findings, including a limit in the generalizability of the findings to a broader population, the diagnosis of MFS was based on the Ghent II criteria without genetic identification of the FBN1 mutation leading to potential bias of diagnosis accuracy as other connective tissue disorder can be misdiagnosed or an incomplete characterization of MFS could be made, and the incorporation of government data to track out-of-hospital mortality can affect the accuracy and completeness of data as it is a secondary source of information.

Furthermore, drawing definitive conclusions about cause-and-effect relationships is inappropriate and provides a challenge for the future. Prospective studies will significantly contribute to a better understanding of managing patients with MFS aortic disease and could lead to more evidence-based decisions and improved patient outcomes.

## Conclusions

Our retrospective case series study sheds light on MFS aortic disease within a middle-income country. The challenges identified, such as the high frequency of emergent procedures and the associated increased risk of reinterventions, highlight the critical role of prompt diagnosis, comprehensive management strategies, and educational initiatives. Our findings emphasize the imperative for healthcare providers to engage in continuous formation and for patients to actively participate in post-treatment programs, particularly in resource-constrained settings. This study serves as a steppingstone, providing a foundation for future investigations. Prospective studies will be pivotal in refining our understanding, validating the observed patterns, and guiding evidence-based decision-making.

## Data Availability

No datasets were generated or analysed during the current study.

## Abbreviations

MFS: Marfan Syndrome.
FBN1: gene for fibrillin-1
ADD: Acute Aortic Dissection
VSRR: valve-sparing root replacement
AHA: American Heart Association
BMI: Body mass index
ASA: American Society of Anesthesiologists
ICU: Intensive Care Unit
SMC: smooth muscle cells
FET: Frozen Elephant Trunk
ET: Elephant Trunk
TEVAR: Endovascular Repair or the Thoracic Aorta
